# Induction of phytoalexins and proteins related to pathogenesis in plants treated with extracts of cutaneous secretions of southern Amazonian Bufonidae amphibians

**DOI:** 10.1371/journal.pone.0211020

**Published:** 2019-01-17

**Authors:** Livia Deice Raasch-Fernandes, Solange Maria Bonaldo, Domingos de Jesus Rodrigues, Gerardo Magela Vieira-Junior, Kátia Regina Freitas Schwan-Estrada, Camila Rocco da Silva, Ana Gabriela Araújo Verçosa, Daiane Lopes de Oliveira, Bryan Wender Debiasi

**Affiliations:** 1 Postgraduate Program in Environmental Sciences, Federal University of Mato Grosso, Sinop, Mato Grosso State, Brazil; 2 Federal University of Mato Grosso and the Postgraduate Program in Environmental Sciences, Sinop, Mato Grosso State, Brazil; 3 Federal University of Piauí, Ministro Petrônio Portela Campus, Teresina, Piauí State, Brazil; 4 State University of Maringá, Maringá, Paraná State, Brazil; 5 Graduate Program in Agronomy, State University of Maringá, Maringá, Paraná State, Brazil; 6 Institute of Agrarian and Environmental Sciences, Federal University of Mato Grosso, Sinop, Mato Grosso State, Brazil; 7 Institute of Health Sciences, Federal University of Mato Grosso, Sinop, Mato Grosso State, Brazil; Universitat Trier, GERMANY

## Abstract

Cutaneous secretions produced by amphibians of the family Bufonidae are rich sources of bioactive compounds that can be useful as new chemical templates for agrochemicals. In crop protection, the use of elicitors to induce responses offers the prospect of durable, broad-spectrum disease control using the plant’s own resistance. Therefore, we evaluated the potential of methanolic extracts of cutaneous secretions of two species of amphibians of the family Bufonidae found in the Amazon biome—*Rhaebo guttatus* (species 1) and *Rhinella marina* (species 2)—in the synthesis of phytoalexins in soybean cotyledons, bean hypocotyls, and sorghum mesocotyls. Additionally, changes in the enzyme activity of β-1,3-glucanase, peroxidase (POX), and polyphenol oxidase (PPO) and in the total protein content of soybean cotyledons were determined. In the soybean cultivar ‘TMG 132 RR’, our results indicated that the methanolic extract of *R*. *guttatus* cutaneous secretions suppressed glyceollin synthesis and β-1,3-glucanase activity and increased POX and PPO activities at higher concentrations and total protein content at a concentration of 0.2 mg/mL. On the other hand, the methanolic extract of *R*. *marina* cutaneous secretions induced glyceollin synthesis in the soybean cultivars ‘TMG 132 RR’ and ‘Monsoy 8372 IPRO’ at 0.1–0.2 mg/mL and 0.2 mg/mL, respectively. The methanolic extract of *R*. *marina* cutaneous secretions also increased the specific activity of POX and PPO in ‘Monsoy 8372 IPRO’ and ‘TMG 132 RR’, respectively, and decreased the activity of β-1,3-glucanases in ‘Monsoy 8372 IPRO’. At 0.3 mg/mL, it stimulated phaseolin synthesis. The extracts did not express bioactivity in the synthesis of deoxyanthocyanidins in sorghum mesocotyls. The study in soybean suggests that the bioactivity in defense responses is influenced by cultivar genotypes. Therefore, these results provide evidence that extracts of cutaneous secretions of these amphibians species may contribute to the bioactivity of defense metabolites in plants.

## Introduction

Chemical control is a nearly indispensable tool in the management of cultivated plant diseases and pests. The continuous application of non-selective synthetic fungicides and pesticides on food crops has been a growing global concern because of the potential deleterious effects on human health and the environment [1]. Moreover, the indiscriminate and excessive use of fungicides on crops has been a major cause of the development of resistant pathogen populations, resulting in the use of higher concentrations of these antifungals and the consequent increase in toxic residues in food products [2]. To ameliorate this situation, researchers are actively investigating alternative methods of disease and pest control.

One of these alternative methods is the utilization of elicitors, compounds that can stimulate the natural defense mechanisms of plants [1]. Induced defense involves the activation of latent resistance mechanisms in plants in response to prior treatment with biotic and abiotic agents [3]. Induction responses may include the synthesis and accumulation of phytoalexins (fungitoxic compounds) [4] and pathogenesis-related proteins (PRs) [5]. These compounds induce biochemical and physiological changes in plants during defense responses and protect against subsequent infections by pathogens [6].

Phytoalexins are antimicrobial compounds, which also exhibit phytotoxic activities [7]. In addition to their fungistatic action, phytoalexins can exert some fungitoxic activities, resulting in cytological abnormalities in fungal cells [8]. The mode of action of phytoalexins on fungi includes cytoplasmic granulation, disorganization of cellular contents, rupture of the plasma membrane, and inhibition of fungal enzymes, leading to the inhibition of spore germination and germ tube elongation and the reduction or inhibition of mycelial growth [9].

Phytoalexins exhibit enormous chemical diversity. Some pterocarpan phytoalexins produced by plants of the family Leguminosae are especially well known: pisatin (*Pisum sativum*), phaseollin (*Phaseolus vulgaris*), and glyceollin (*Glycine max*) [10]. The main phytoalexins of the family Poaceae (*Oryza sativa*, *Zea mays*, and *Sorghum bicolor*) are represented by members of the labdane-related diterpenoid superfamily (zealexins, kauralexins, momilactones, oryzalexins, and phytocassanes) [11, 12], flavanones, 3-deoxyanthocyanidins (an unusual group of flavonoid phytoalexins) [13], and phenylamides [14].

PRs are induced in the host in response to pathogen infection or abiotic stimuli, and their accumulation is an integral component of innate immune responses in plants [15]. PRs not only accumulate locally in the infected leaf but also associate with the development of a hypersensitive response or systemic acquired resistance against infection by microorganisms [16]. PRs include enzymes, such as chitinases (CHI), β-1,3-glucanases (GLU), phenylalanine ammonia-lyase (PAL) [17], peroxidase (POX), and polyphenol oxidase (PPO) [18–21], and may act directly on the pathogen (via the inhibition of microbial growth or spore germination) or indirectly (via the induction of host resistance) [22].

Extensive reports are available on the induction of resistance in plants through the direct and indirect involvement of elicitors including extracts and essential oils of plants [23], basidiocarp extracts, and synthetic chemicals (aminobutyric acid, salicylic acid, jasmonic acid, and acibenzolar-S-methyl) [24]. In particular, studies on phytoalexin induction include inductions of glyceollin by *Equisetum* sp. preparations [25] and by aqueous extracts of basidiocarps of *Agaricus blazei*, *Lentinula edodes*, and *Pycnoporus sanguineus* [26]; glyceollins and deoxyanthocyanidins by crude aqueous extracts and tinctures of *Ruta graveolens*, *Origanum majorana*, *Baccharis trimera* [27], *Hymenolobium petraeum*, *Qualea albiflora*, and *Corymbia citriodora* [28]; and phaseolins by essential oil of *Eucalyptus globulus* [29], homeopathic preparations of *C*. *citriodora* and *Calcarea carbonica* [30], and salicylic acid [1].

Paula et al. [31] evaluated the extract and fractions of leaves of *Bauhinia ungulata* and observed changes in the synthesis of α-amylase, POX, catalase, and PPO in lettuce (*Lactuca sativa*) seedlings for at least one of the tested concentrations. Curvêlo et al. [32] found that silicon (Si) affected the activity of POX, PPO, CHI, GLU, and PAL and increased the resistance of cotton plants to *Ramularia* leaf spot (*Ramularia areola*).

These results indicate that several natural products may elicit action, and the varied chemical nature of the elicitors demonstrates that no single structural characteristic dictates the biological activity. Their biological relevance and structural diversity [33] make natural products good starting points in the identification of new elicitors for crop protection. According to Atanasov et al. [34], since natural products are made from living organisms, they possess properties that are evolutionarily optimized for serving different biological functions developed during biosynthesis with superior chemical diversity and complexity to synthetic chemicals.

Structural diversity, however, is not the only reason why natural products are of interest for drug development. An important additional feature is that they often possess highly selective and specific biological activities based on mechanisms of action [33, 35]. Thus, over the years, natural products have delivered a significant number of valuable starting points and some of the most important modes of action in agrochemical development [35, 36]. For example, spinosyns [35, 36], afidopyropen [37], fenpicoxamid [38], and strobilurins are synthetic pyrethroids that are reconfigurations of the core structure of the natural product (synthetic mimic) [35, 36].

Substances with as yet unknown potential as agrochemicals are present in cutaneous secretions of amphibians, especially frogs of the family Bufonidae, whose glands synthesize a wide range of bioactive secondary metabolites of different chemotypes [39–41]. These molecules play a crucial role in the physiological functions of these animals, especially for predation and protection against microorganisms [42]. Usually, these secretions contain a wide range of molecules, such as biogenic amines, alkaloids, steroids, peptides, and high-molecular-weight proteins [43–45].

Therefore, we evaluated the potential of methanolic extracts of cutaneous secretions of two amphibian species of the family Bufonidae found in the Amazon biome—*Rhaebo guttatus* (species 1) and *Rhinella marina* (species 2)—for the induction of phytoalexins in soybean (*G*. *max*) cotyledons, bean (*P*. *vulgaris*) hypocotyls, and sorghum (*S*. *bicolor*) mesocotyls, as well as changes in the activity of GLU, POX, and PPO, and in the total protein content of soybean cotyledons.

## Materials and methods

### Sample collection and preparation

We used the cutaneous secretions of two amphibian species of the family Bufonidae—*R*. *guttatus* (species 1) and *R*. *marina* (species 2)—from southern Amazon. The cutaneous secretions were collected by a team of biologists from the Federal University of Mato Grosso (Universidade Federal de Mato Grosso), Sinop Campus, Mato Grosso state, Brazil (IBAMA collection permit No. 30034–1).

Methanolic extracts were obtained by drying the cutaneous secretions in silica gel, extracting the samples three times in methanol, and evaporating the extracts in a rotary evaporator. The crude extracts were weighed, diluted in sterile water to a concentration of 0.8 mg/mL, filtered through a Millipore membrane (0.22 μm), and diluted to the desired concentrations. The methanolic extracts of *R*. *guttatus* or *R*. *marina* (at concentrations of 0.1, 0.2, 0.3, 0.4, or 0.5 mg/mL), sterile water (negative control), and *Saccharomyces cerevisiae* (20%) from Fleischmann’s commercial yeast (positive control) were used in the treatments.

In this study, we selected three crops (soybean, beans, and sorghum) that are produced in the central region of Brazil and adapted to local edaphoclimatic conditions. The phytoalexin assays were performed in triplicate, with seven treatments per assay and five replications per treatment. Samples of soybean cotyledons were wrapped in aluminum foil and stored at −20°C for subsequent analysis of enzyme activity. The samples of cotyledons were macerated in liquid nitrogen and homogenized in 4 mL of 50 mM potassium phosphate buffer (pH 7.0) containing 0.1 mM EDTA and 1% (w/w) PVP (polyvinylpyrrolidone) in a porcelain crucible. The homogenate was centrifuged for 30 min at 14,500 g at 4°C, and the supernatant obtained, considered as the enzyme extract, was stored at −20°C. The extract was used for the determination of POX, PPO, protein concentration using the Bradford method [46], and GLU [47].

### Phytoalexin assays in soybeans

Soybean seeds, used by the producers in the central region of Brazil, were selected. After selecting the main soybean cultivars, we opted to use different genetic varieties—‘Monsoy 8372 IPRO’ (transgenic RR2), ‘TMG 132 RR’ (transgenic RR1), and ‘TMG 4182’ (nontransgenic)—to determine variability in glyceollin induction in each cultivar genotype. Seeds of the three cultivars were sown in sterilized sand and germinated at ambient temperature (22°C–28°C). After 7 days, the cotyledons were removed from the seedlings, washed with distilled water, dried, weighed, and cut into sections from the lower extremity with a thickness of approximately 1 mm and a diameter of approximately 6 mm. The cotyledons were weighed and placed in petri dishes containing filter paper moistened with sterile water, and five cotyledons were used in each replicate. Each petri dish constituted one replicate. A 75-μL aliquot of each treatment solution was applied on each cotyledon. The petri dishes were maintained at 25°C in the dark for 20 h. After this period, the cotyledons were transferred to test tubes containing 15 mL of sterile water and agitated for 1 h for glyceollin extraction. Absorbance was read at 285 nm using a UV–visible spectrophotometer (200–1000 nm; EDUTEC/EEQ9011B.UV-B, Astral Scientific, Brazil) [48]. The data were expressed as absorbance per gram of fresh tissue (Abs gtf^−1^).

### Phytoalexin assays in beans

Phytoalexin induction in beans was determined according to the methodology proposed by Dixon et al. [49] with some modifications. Bean seeds (ANFc 9) were disinfected with 1% bleach for 5 min, rinsed in sterile water, sown in sterilized sand, and grown in an incubator in the dark. After 6 days, sections with a thickness of approximately 5 cm were cut from etiolated hypocotyls of seedlings, washed with sterile water, and dried on absorbent paper. The hypocotyl segments were transferred to petri dishes containing filter paper moistened with sterile water, and a 1-mL aliquot of each treatment/replicate was applied to each dish. Four hypocotyl segments were used in each replicate, and each petri dish constituted one replicate. The petri dishes were maintained at 25°C in the dark for 48 h. The hypocotyls were then transferred to test tubes containing 10 mL of ethanol and maintained at 4°C for 48 h. After this period, the tubes were agitated for 1 h for phaseolin extraction, and absorbance was read at 280 nm for the quantification of phaseolins. The data were expressed as Abs gtf^−1^.

### Phytoalexin assays in sorghum

Sorghum seeds (hybrid A9735R; Nidera) were treated with dicarboximide (Captan 200FS), disinfected with 1% bleach for 15 min, rinsed in sterile water, and soaked in water at room temperature (22°C–28°C) for 24 h. After this period, the seeds were wrapped in sheets of moistened germination paper and incubated in the dark at 28°C for approximately 6 days. The elongation of the mesocotyls was interrupted by exposing the produced seedlings to light for 4 h. The mesocotyls were excised and transferred to a test tube (three mesocotyls per replicate) containing 1 mL of each treatment/replicate and maintained in a humid chamber at 25°C under fluorescent light for 60 h [50]. Each test tube constituted one replicate. Subsequently, the mesocotyls were removed from the test tubes, and their upper extremities were dried, weighed, and cut into small segments. The obtained segments were placed in microcentrifuge tubes containing 1.4 mL of 80% acidified methanol (0.1% HCl; v/v) and maintained at 4°C for 96 h. Absorbance was read at 480 nm for the quantification of deoxyanthocyanidins. The data were expressed as Abs gtf^−1^.

### Quantification of POX activity

POX activity was quantified by measuring the conversion of guaiacol to tetraguaiacol after mixing 20 μL of the enzyme extract with 2.9 mL of the substrate (80 μL of guaiacol, 122.4 μL of peroxide, and 40 mL of 0.01 M phosphate buffer, pH 6.0) [51]. POX activity was determined at 30°C by reading the absorbance at 470 nm for 2 min at 10-s intervals using a spectrophotometer. The specific activity was expressed in units of absorbance min^−1^ mg^−1^ of protein [46].

### Quantification of PPO activity

PPO activity was determined according to the method of Duangmal and Apenten [52] with modifications and consisted of the quantification of the oxidative conversion of catechol to quinone. The enzyme extract contained 12 mL of polyphenol buffer and 0.0264 g of catechol and was maintained in a water bath at 30°C for 30 min. The substrate preparation contained 20 mM catechol dissolved in 100 mM potassium phosphate buffer (pH 6.8). The reaction solution was obtained by mixing 980 μL of the substrate with 20 μL of the enzyme extract. The reaction was performed at 30°C and quantified by reading the absorbance at 420 nm for 2 min using a spectrophotometer. The proteins were quantified according to Bradford [46] and expressed as absorbance min^−1^ mg^−1^ of protein.

### Quantification of GLU

The activity of GLU was determined by quantifying reducing sugars released by the hydrolysis of the substrate laminarin using Vogelsang and Barz’s method [47]. The reaction volume contained 30 μL of the enzyme extract, 120 μL of the phosphate extraction buffer, and 150 μL of laminarin (2 mg/mL) prepared in 0.01 M phosphate buffer solution (pH 6.0). For the control, laminarin was added to the same reaction volume immediately before the quantification of sugars (without incubation). The reaction was conducted at 40°C for 1 h in a water bath. Subsequently, a 30-μL aliquot was removed from the test tubes and 1.5 mL of 0.5% p-hydroxybenzoic acid hydrazide prepared in 0.5 M NaOH was added. The mixture was maintained at 100°C for 5 min and cooled in an ice bath. Absorbance was read at 410 nm using a spectrophotometer, and the absorbance of the control was deducted from the absorbance of the samples. For the quantification of sugars, a standard curve representing different concentrations of glucose was used. The values were expressed as equivalent mg of glucose h^−1^ mg^−1^ protein.

### Quantification of total protein content

Total protein was quantified using the method proposed by Bradford [46]. The reaction volume contained 50 μL of the enzyme extract and 2.5 mL of Bradford reagent, and the test tubes were mixed by vortexing. A volume of 50 μL of distilled water and 2.5 mL of Bradford reagent were used as a reference. After incubation for 5 min, absorbance was measured at 595 nm using a spectrophotometer. The absorbance of the samples was compared with the absorbance values of a standard curve containing 0–4.0 mg/mL of bovine serum albumin. Protein concentration was expressed as mg protein mL^−1^ of fresh weight.

### Statistical analyses

The experiment used a completely randomized design, and the data were analyzed using the program SISVAR version 4.3 [53]. For phytoalexin assays, the results were subjected to analysis of variance (ANOVA) using the F-test, and the means were compared using the Scott–Knott test (P ≤ 0.05). For the evaluation of enzyme activity, the results were subjected to ANOVA using the F-test and when significant, were subjected to regression analysis (P ≤ 0.05), adjusting the regression equations (R^2^). Treatment with *S*. *cerevisiae* was used as an additional control [54].

## Results

### Synthesis of glyceollins in soybean cotyledons

**Fig 1:**
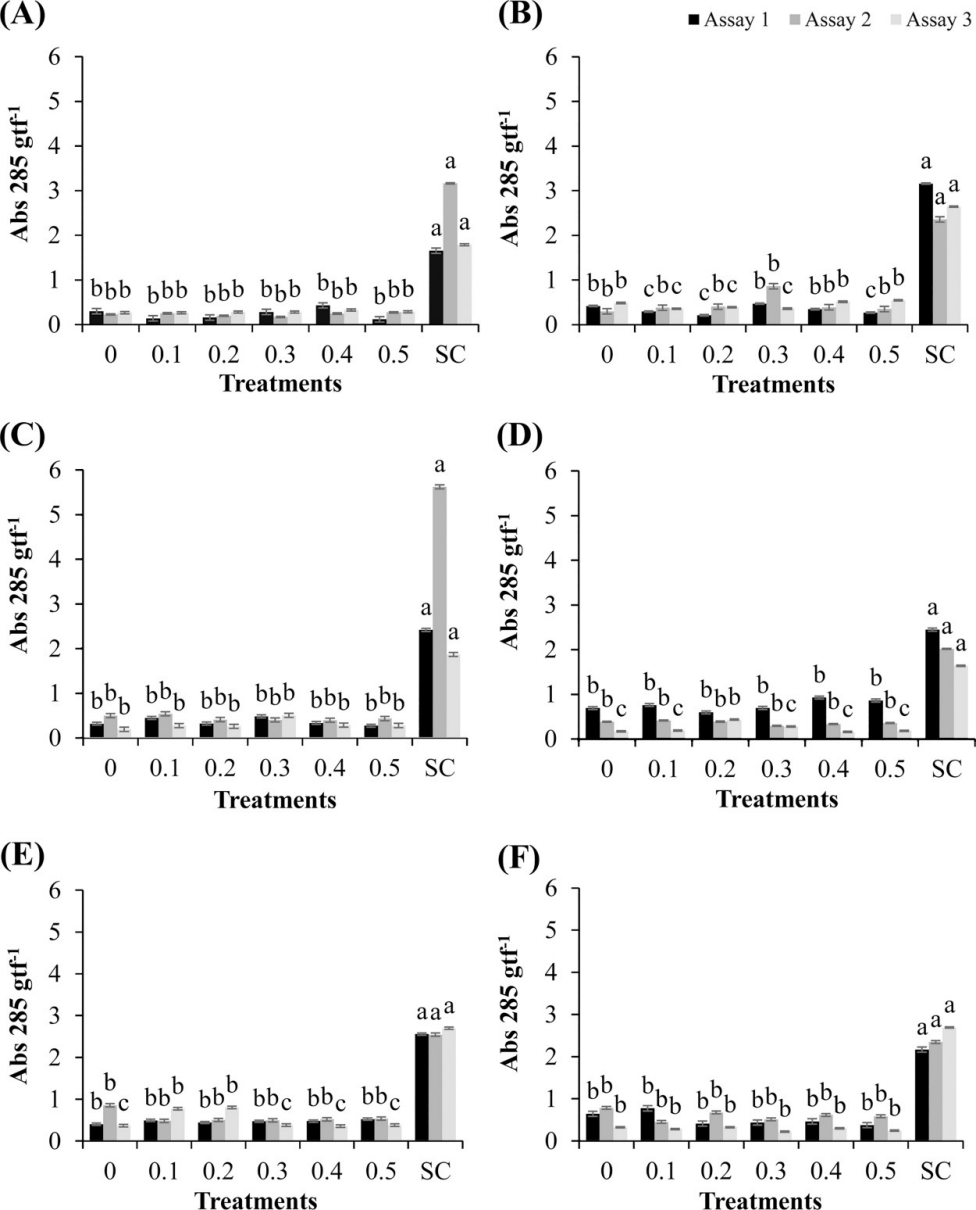
Synthesis of glyceollins in soybean cotyledons treated with different concentrations of *R*. *guttatus* and *R*. *marina* (0.1, 0.2, 0.3, 0.4, and 0.5 mg/mL), sterile water (0), and *S*. *cerevisiae* (SC). Cultivars treated with *R*. *guttatus* cutaneous secretions (species 1): (**A**) ‘Monsoy 8372 IPRO’; (**B**) ‘TMG 132 RR’; and (**C**) nontransgenic ‘TMG 4182’. Cultivars treated with *R*. *marina* cutaneous secretions (species 2): (**D**) ‘Monsoy 8372 IPRO’; (**E**) ‘TMG 132 RR’; and (**F**) nontransgenic ‘TMG 4182’. The experiments were performed three times for each treatment. The same letters indicate absence of significant differences by the Scott–Knott test at P ≤ 0.05. Metric bars indicate the standard error of the mean (SE). Data were transformed as follows: (*x*+1)^0.5^.

The responses of the soybean cultivars following the application of the methanolic extract of *R*. *guttatus* (species 1) and *R*. *marina* (species 2) cutaneous secretions and their respective synthesis of glyceollins are shown in Fig 1. As shown in Fig 1B, the cultivar ‘TMG 132 RR’ exhibited inhibitory activity in glyceollin synthesis in the cotyledons on treatment with the methanolic extract of *R*. *guttatus* cutaneous secretions at concentrations 0.1, 0.2, and 0.5 mg/mL and 0.1, 0.2, and 0.3 mg/mL, in assays 1 and 3, respectively (P ≤ 0.05), with respect to the negative control. These natural toxins produced by soybean are important for the interaction of this leguminous plant with phytopathogens. The phytoalexins have cytological effects, which culminate in growth inhibition or death of the pathogen. This observation is important because it suggests that the cultivar exhibits enhanced disease susceptibility on treatment with this extract. In the cultivars ‘Monsoy 8372 IPRO’ and nontransgenic ‘TMG 4182’, an increase was observed only on treatment with *S*. *cerevisiae*, which stimulated glyceollin synthesis (Fig 1A and 1C).

The methanolic extract of cutaneous secretions of *R*. *marina* promoted glyceollin synthesis at the concentration of 0.2 mg/mL (assay 3) in ‘Monsoy 8372 IPRO’ (Fig 1D) and at the concentrations of 0.1 and 0.2 mg/mL in ‘TMG 132 RR’ (Fig 1E), and the obtained values were significantly greater (P ≤ 0.05) than those with the negative control (sterile water). Treatment with *S*. *cerevisiae* significantly increased glyceollin synthesis in all cultivars in the three independent assays. Intriguingly, the responses of glyceollin synthesis were different only in cultivars with modified genotype treated with the extracts. In this respect, ‘TMG 132 RR’ belongs to the first group of transgenic cultivars (RR1), with resistance to glyphosate, whereas ‘Monsoy 8372 IPRO’ belongs to the second generation of transgenic cultivars (RR2), with tolerance to glyphosate and resistance to caterpillars.

### Synthesis of phaseolins in bean hypocotyls and deoxyanthocyanidins in sorghum mesocotyls

**Fig 2:**
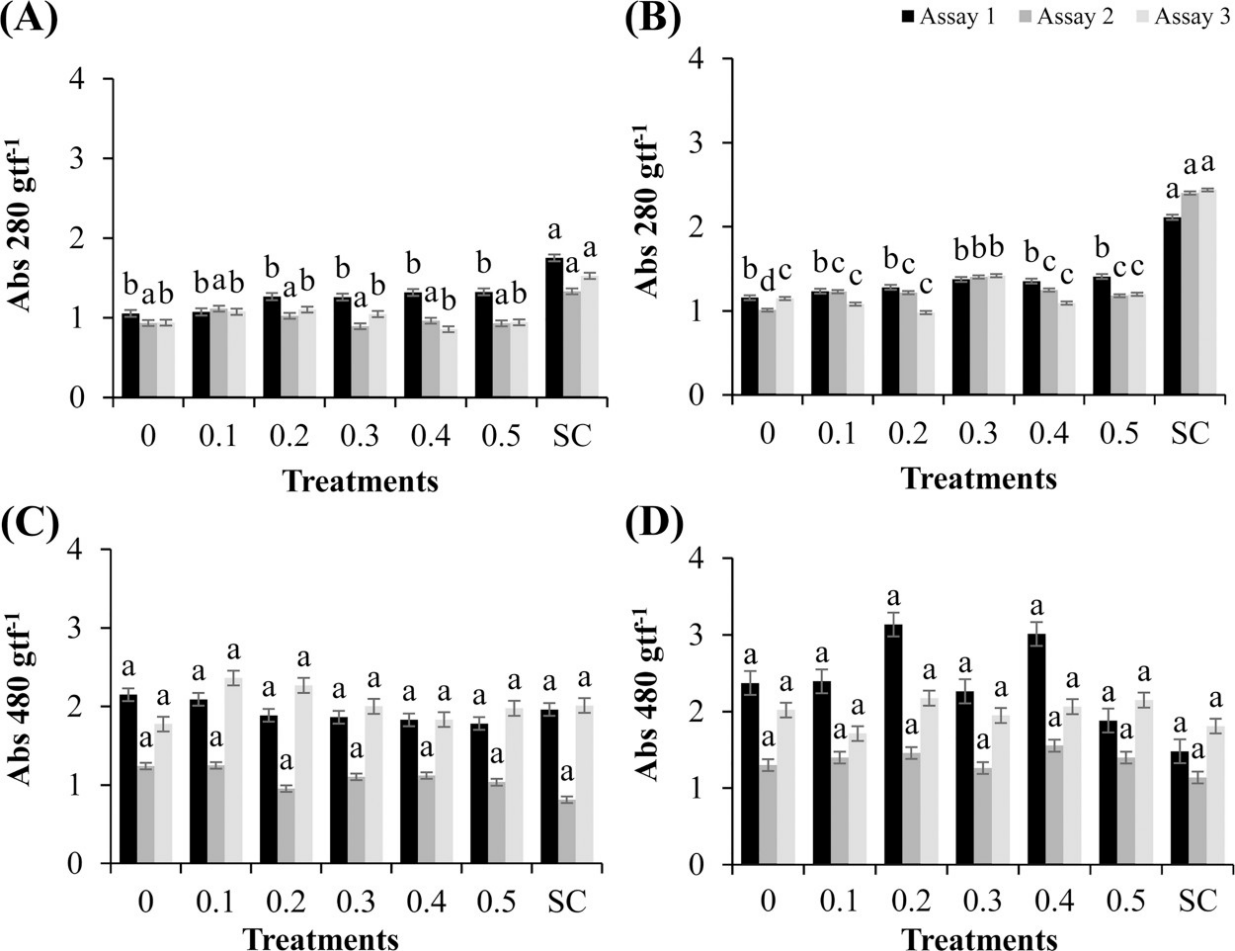
Synthesis of phaseolins in bean hypocotyls and deoxyanthocyanidins in sorghum mesocotyls treated with different concentrations of *R*. *guttatus* and *R*. *marina* (0.1, 0.2, 0.3, 0.4, and 0.5 mg/mL), sterile water (0), and *S*. *cerevisiae* (SC). (**A**) Bean hypocotyls treated with *R*. *guttatus* cutaneous secretions; (**B**) Bean hypocotyls treated with *R*. *marina* cutaneous secretions; (**C**) Sorghum mesocotyls treated with *R*. *guttatus* cutaneous secretions; and (**D**) Sorghum mesocotyls treated with *R*. *marina* cutaneous secretions. The experiments were performed three times for each treatment. The same letters indicate absence of significant differences by the Scott–Knott test at P ≤ 0.05. Metric bars indicate the standard error of the mean (SE). Data were transformed as follows: (*x*+1)^0.5^.

No significant synthesis of phaseolins was observed in bean hypocotyls treated with the methanolic extract of *R*. *guttatus* cutaneous secretions, and only the influence in the positive control in assays 1 and 3 was verified (P ≤ 0.05), with the mean levels of phytoalexins being 1.75 and 1.53 Abs 280 gpf^−1^, respectively (Fig 2A).

The methanolic extract of *R*. *marina* cutaneous secretions in assay 2 (Fig 2B) significantly increased phaseolin synthesis at all tested concentrations compared with the negative control (P ≤ 0.05). The mean phytoalexin synthesis rate was significantly higher at the concentration of 0.3 mg/mL (1.40 Abs 280 gpf^−1^) than those at the other concentrations of 0.4, 0.1, 0.2, and 0.5 mg/mL (1.25, 1.23, 1.22, and 1.18 Abs 280 gpf^−1^, respectively) without significant differences between these values.

In addition, the methanolic extract of *R*. *marina* cutaneous secretions significantly increased the phaseolin synthesis in bean hypocotyls at the concentration of 0.3 mg/mL in assay 3, with a mean level of 1.42 Abs 280 gpf^−1^, which was significantly higher than that of the negative control, which had a mean level of 1.15 Abs 280 gpf^−1^. The observation results showed the presence of molecules in the composition of this extract that induced defense response in the beans. This fact indicated the potential of this extract in the protection of this culture against phytopathogens.

Treatment with *S*. *cerevisiae* significantly increased phaseolin synthesis in the three tested assays compared with the other treatments, with mean levels of 2.11, 2.40, and 2.44 Abs 280 gpf^−1^, respectively. The methanolic extract of *R*. *guttatus* (species 1) and *R*. *marina* (species 2) cutaneous secretions did not significantly affect the synthesis of deoxyanthocyanidins in sorghum mesocotyls compared with that in the negative control (Fig 2C and 2D).

### Activity of enzymes (POX, PPO, and GLU) and total protein content

**Fig 3:**
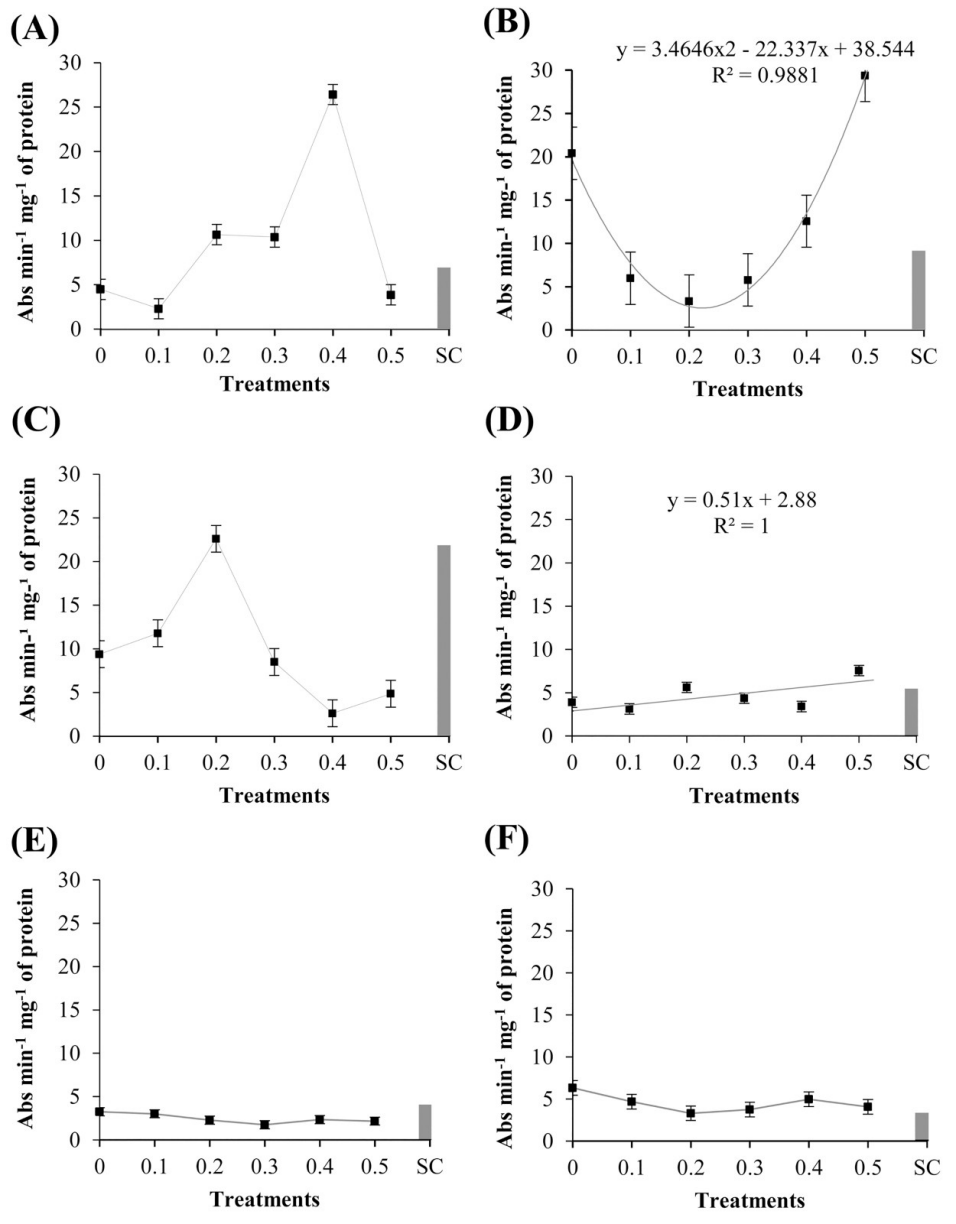
Specific activity of peroxidases in ‘Monsoy 8372 IPRO,’ ‘TMG 132 RR,’ and nontransgenic ‘TMG 4182’ treated with different concentrations of *R*. *guttatus* and *R*. *marina* (0.1, 0.2, 0.3, 0.4, and 0.5 mg/mL), sterile water (0), and *S*. *cerevisiae* (SC). Cultivars treated with *R*. *guttatus* cutaneous secretions (species 1): (**A**) ‘Monsoy 8372 IPRO’; (**B**) ‘TMG 132 RR’; and (**C**) nontransgenic ‘TMG 4182’. Cultivars treated with *R*. *marina* cutaneous secretions (species 2): (**D**) ‘Monsoy 8372 IPRO’; (**E**) ‘TMG 132 RR’; and (**F**) nontransgenic ‘TMG 4182’. Data are the mean of the three independent experiments. Data when significant were subjected to regression analysis (P ≤ 0.05), adjusting the regression equations (R^2^). Treatment with *S*. *cerevisiae* was used as an additional control. Metric bars indicate the standard error of the mean (SE).

The cotyledons of ‘TMG 132 RR’ treated with the methanolic extract of *R*. *guttatus* cutaneous secretions were significantly different (P ≤ 0.05) in the activity of guaiacol POX (Fig 3B). The enzyme activity was reduced in the concentrations 0.1–0.4 mg/mL, when compared with the control negative (sterile water). The extract concentration of 0.5 mg/mL showed a higher activity of guaiacol POX, differing in this respect from the other treatments (P ≤ 0.05). This shows that the extract concentrations have different dosage of active and inactive molecules, and this elicitor-mediated induction is related to the higher concentration of these substances. The activity of guaiacol POX in ‘Monsoy 8372 IPRO’ significantly differ among the treatments with the methanolic extract of *R*. *marina* cutaneous secretions (P ≤ 0.05), as can be verified in Fig 3D. The activity of POX was higher at the extract concentrations of 0.2 and 0.5 mg/mL, differing from the other treatments.

**Fig 4:**
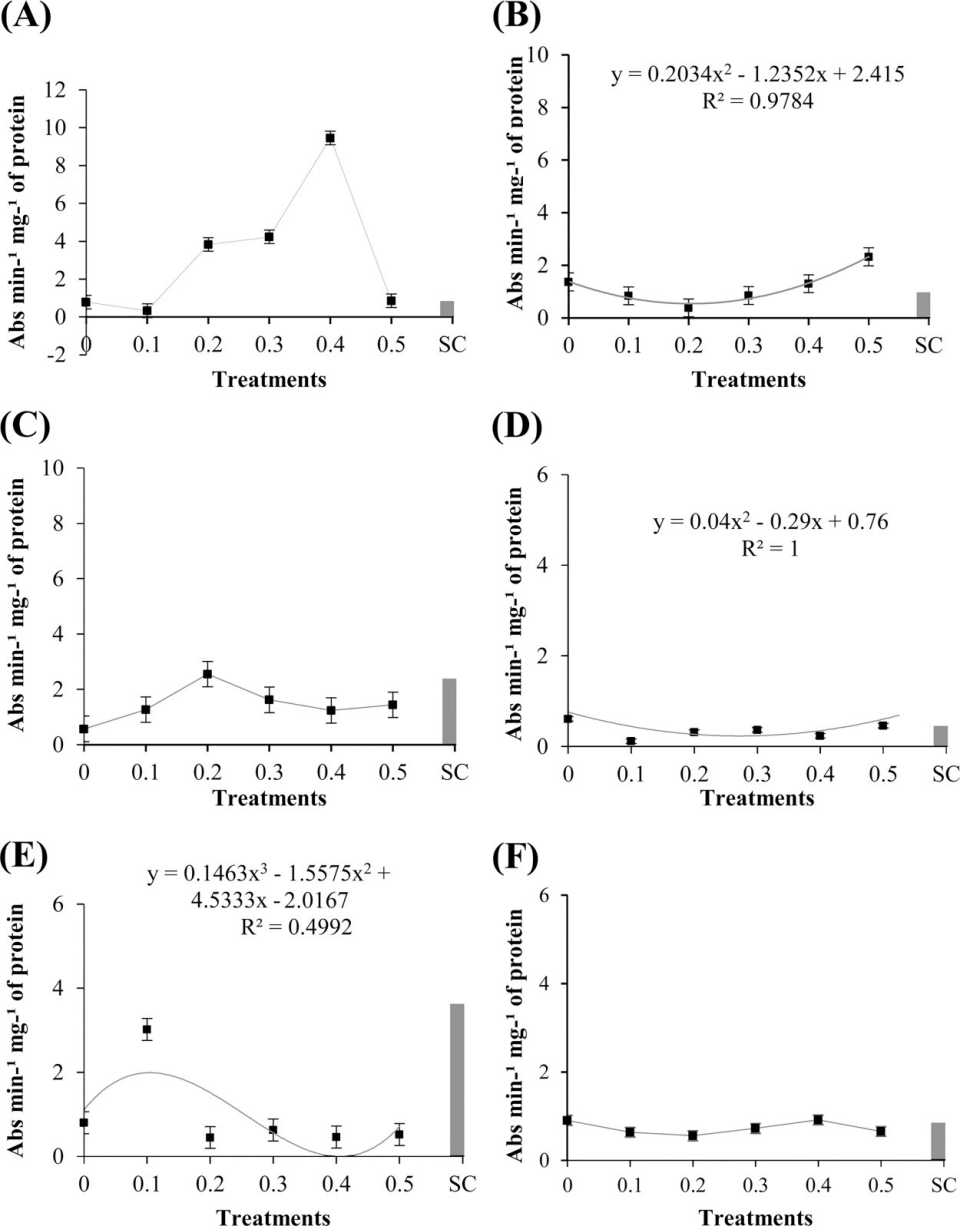
Specific activity of polyphenol oxidases in ‘Monsoy 8372 IPRO,’ ‘TMG 132 RR,’ and nontransgenic ‘TMG 4182’ treated with different concentrations of *R*. *guttatus* and *R*. *marina* (0.1, 0.2, 0.3, 0.4, and 0.5 mg/mL), sterile water (0), and *S*. *cerevisiae* (SC). Cultivars treated with *R*. *guttatus* cutaneous secretions (species 1): (**A**) ‘Monsoy 8372 IPRO’; (**B**) ‘TMG 132 RR’; and (**C**) nontransgenic ‘TMG 4182’. Cultivars treated with *R*. *marina* cutaneous secretions (species 2): (**D**) ‘Monsoy 8372 IPRO’; (**E**) ‘TMG 132 RR’; and (**F**) nontransgenic ‘TMG 4182’. Data were the mean of three independent experiments. Data when significant were subjected to regression analysis (P ≤ 0.05), adjusting the regression equations (R^2^). Treatment with *S*. *cerevisiae* was used as an additional control. Metric bars indicate the standard error of the mean (SE).

The cotyledons of ‘TMG 132 RR’ treated with the methanolic extract of *R*. *guttatus* cutaneous secretions showed response significant (P ≤ 0.05), as can be verified in Fig 4B. The concentration 0.5 mg/mL increased the activity of PPO, compared with the other treatments. Moreover, the lower concentrations of the extract reduced PPO activity in this cultivar when compared with that of the sterile water. On application of this same extract in ‘TMG 132 RR’, similar responses of POX and PPO activities were observed, which suggests the existence of a correlation between the synthesis of these two enzymes. Further, in both enzymes, the activity increased with increase in the concentration of the extract. This fact suggested the concentration-dependent effect of the extract in the synthesis of POX and PPO.

By comparing the effect of methanolic extract of *R*. *marina* cutaneous secretions, significant difference was observed between the activity of PPO in the cotyledons of ‘Monsoy 8372 IPRO’ and ‘TMG 132 RR’ (P ≤ 0.05) (Fig 4D and 4E). The concentrations of the extract demonstrated reduced PPO activity when compared with that with sterile water. This enzyme is known to induce metabolites in defense mechanisms of plants, and the reduction could increase oxidative stress. In ‘TMG 132 RR’, PPO activity was higher at the extract concentration of 0.1 mg/mL. These data suggest that concentrations higher than 0.1 mg/mL showed no beneficial effects for the cotyledons in this cultivar.

**Fig 5:**
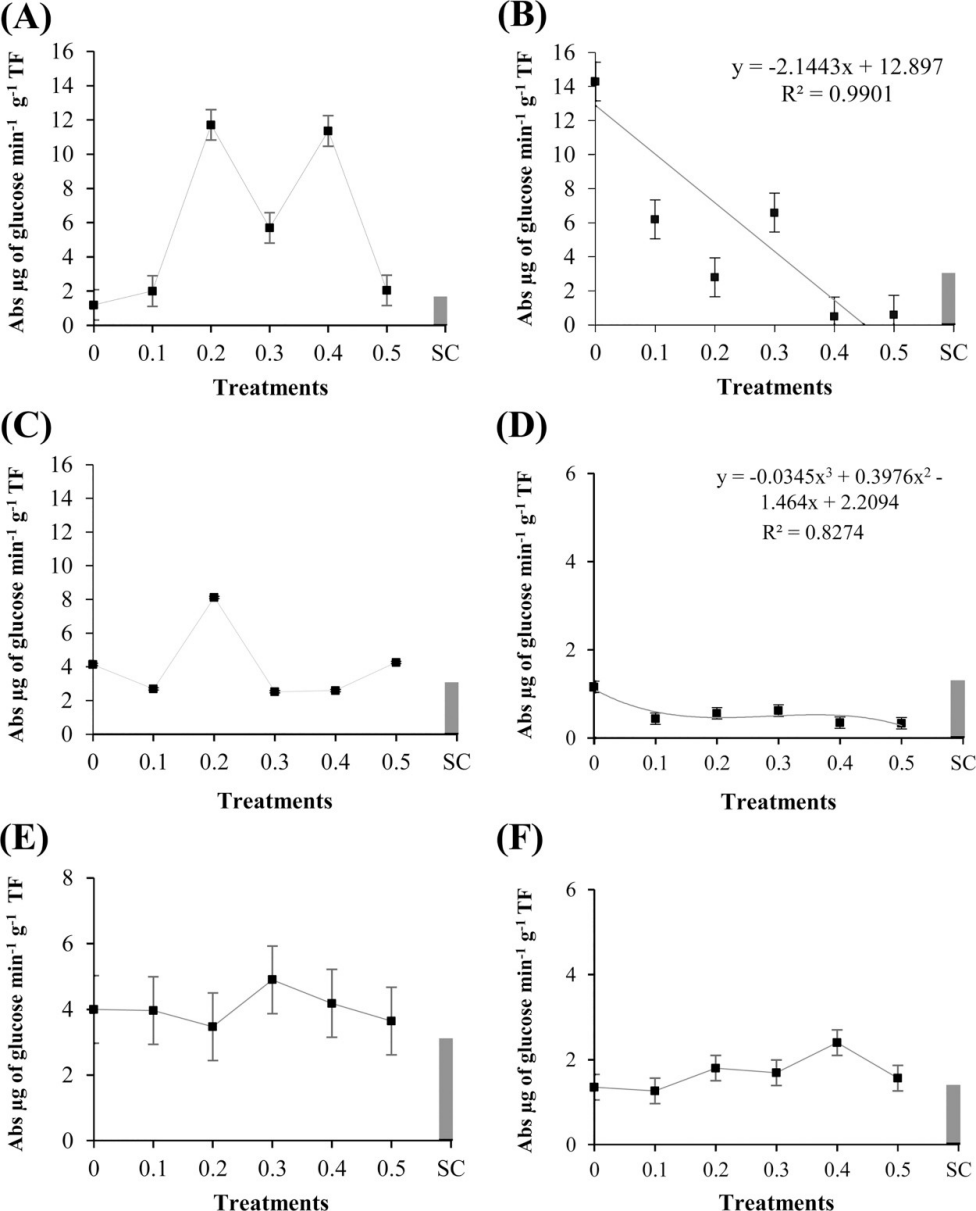
Specific activity of β-1,3-glucanases in ‘Monsoy 8372 IPRO,’ ‘TMG 132 RR,’ and conventional ‘TMG 4182’ exposed to different concentrations of *R*. *guttatus* and *R*. *marina* (0.1, 0.2, 0.3, 0.4, and 0.5 mg/mL), sterile water (0), and *S*. *cerevisiae* (SC). Cultivars treated with the extract of *R*. *guttatus* cutaneous secretions (species 1): (**A**) ‘Monsoy 8372 IPRO’; (**B**) ‘TMG 132 RR’; and (**C**) nontransgenic ‘TMG 4182’. Cultivars treated with the extract of *R*. *marina* cutaneous secretions (species 2): (**D**) ‘Monsoy 8372 IPRO’; (**E**) ‘TMG 132 RR’; and (**F**) nontransgenic ‘TMG 4182’. Data are the mean of three independent experiments. Data when significant were subjected to regression analysis (P ≤ 0.05), adjusting the regression equations (R^2^). Treatment with *S*. *cerevisiae* was used as an additional control. Metric bars indicate the standard error of the mean (SE).

The GLU activity of cotyledons treated with different concentrations of the methanolic extract of *R*. *guttatus* cutaneous secretions were significant (P ≤ 0.05) for ‘TMG 132 RR’ (Fig 5B). Analyses of the concentrations of the extract showed that GLU activity was reduced with the application of the extract of *R*. *guttatus*. In the presence of this extract, GLU activity gradually reduced. These results revealed the differences in the synthesis of GLU compared with that of POX and PPO for this cultivar. Application of a higher concentration of the extract reduced GLU activity, whereas it promoted POX and PPO activities.

The experiments with the methanolic extract of *R*. *marina* cutaneous secretions demonstrated that GLU activity was significantly affected in ‘Monsoy 8372 IPRO’ (Fig 5A). However, the activity of the enzyme with the concentrations of the extract of *R*. *marina* was significantly lower compared with that with sterile water. The activity of this enzyme was reduced with increased concentration of the extract. This tendency was observed in ‘TMG 132 RR’ and ‘Monsoy 8372 IPRO’ with the application of the extracts of *R*. *guttatus* and *R*. *marina*, respectively.

**Fig 6:**
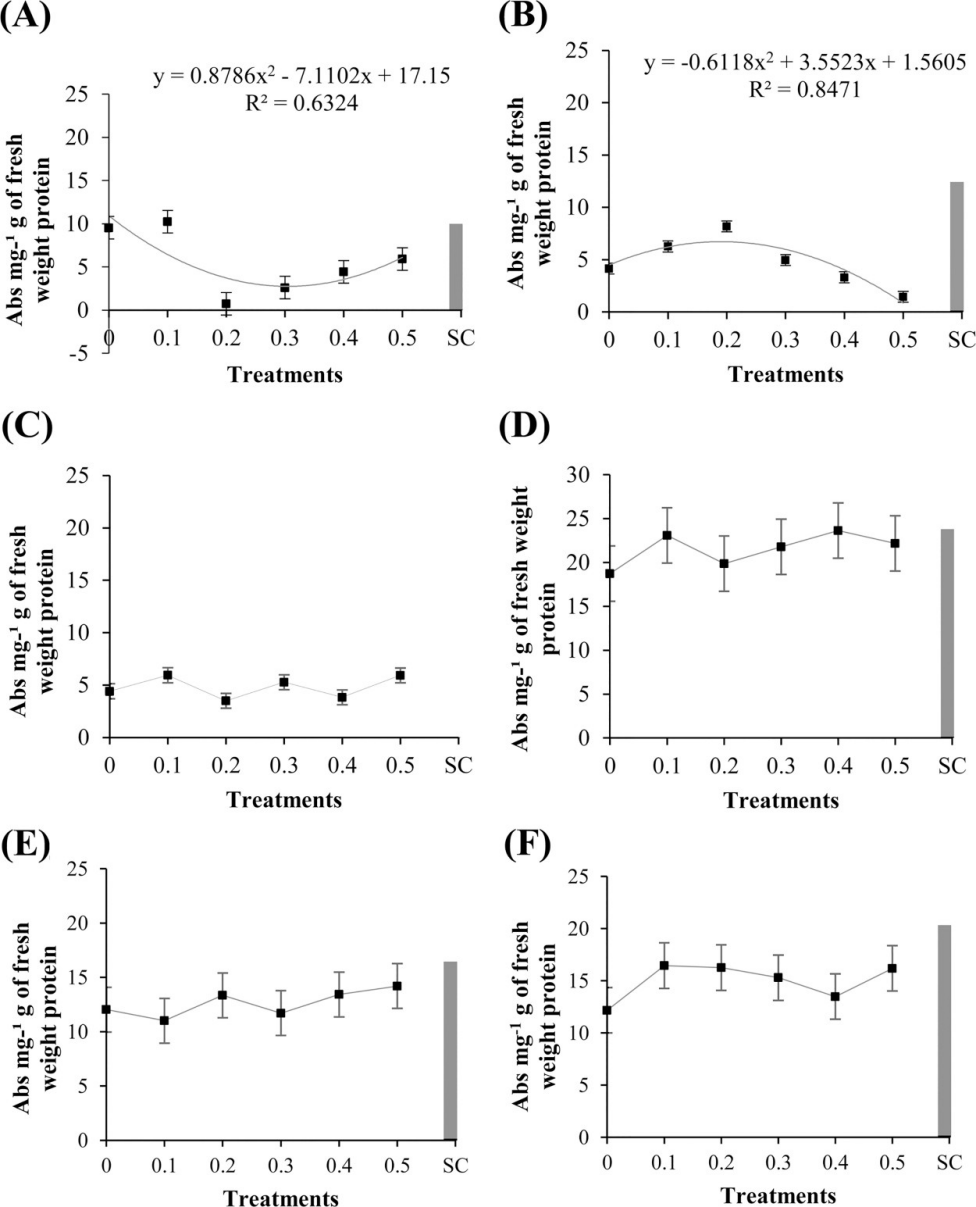
Total protein content in ‘Monsoy 8372 IPRO,’ ‘TMG 132 RR,’ and nontransgenic ‘TMG 4182’ treated with different concentrations of *R*. *guttatus* and *R*. *marina* (0.1, 0.2, 0.3, 0.4, and 0.5 mg/mL), sterile water (0), and *S*. *cerevisiae* (SC). Cultivars treated with *R*. *guttatus* cutaneous secretions (species 1): (**A**) ‘Monsoy 8372 IPRO’; (**B**) ‘TMG 132 RR’; and (**C**) nontransgenic ‘TMG 4182’. Cultivars treated with *R*. *marina* cutaneous secretions (species 2): (**D**) ‘Monsoy 8372 IPRO’; (**E**) ‘TMG 132 RR’; and (**F**) nontransgenic ‘TMG 4182’. Data are the mean of three independent experiments. Data when significant were subjected to regression analysis (P ≤ 0.05), adjusting the regression equations (R^2^). Treatment with *S*. *cerevisiae* was used as an additional control. Metric bars indicate the standard error of the mean (SE).

The total protein content significantly changed (P ≤ 0.05) after the treatment of the soybean cultivars ‘Monsoy 8372 IPRO’ and ‘TMG 132 RR’ with the methanolic extract of *R*. *guttatus* cutaneous secretions, as can be verified in Fig 6A and 6B. Total protein content of ‘Monsoy 8372 IPRO’ was significantly lower on treatment with the various concentrations of the extract than with the negative control (sterile water). For ‘TMG 132 RR’, the total protein content was found to be increased at the concentration of 0.2 mg/mL, following which it gradually decreased. There was no significant difference between the treatments in the assays of the methanolic extract of *R*. *marina* cutaneous secretions (Fig 6D, 6E, and 6F).

## Discussion

### Synthesis of glyceollins in soybean cotyledons

The suppression of glyceollin synthesis in soybean cultivar ‘TMG 132 RR’ by the methanolic extract of cutaneous secretions of species 1 (*R*. *guttatus*) was probably because the plant recognizes this extract as an inducer of susceptibility. This differentiated response may be related to changes in the genome of this cultivar, which contains transgenes that are tolerant to the glyphosate herbicide *Roundup Ready* (RR). This change in the genetic material may have affected the ability of the cultivar to recognize the substance as an elicitor, considering that the defense mechanisms of plants are mediated by *R* genes [55]. The expression of *R* genes might modulate the disease resistance or susceptibility, with outcomes dependent on the genetic constitution of the host [56, 57]. It suggests that host genotypes react differently to an elicitor [58].

Thakur and Sohal [59] reported that the identification of a pathogen typically occurs when avirulence (Avr) gene products, secreted by pathogen, bind to or indirectly interact with the product of a plant resistance (R) gene (gene for gene model). When both the R gene and corresponding Avr genes are present, recognition occurs, which leads to active resistance of the plant and avirulence of the pathogen. If either Avr gene in the pathogen or R gene in the host is absent or is mutated, no recognition will occur and outcome will be compatible reaction and disease.

Some elicitors have the same specificity as the pathogen has with its host [60]. Thus, on account of the high level of specificity, gene for gene system gives a decent structure to decide whether the result of the Avr gene can likewise go about as a race-/cultivar-specific elicitor of defense response like phytoalexin accumulations [58]. If the pathogen does not possess the Avr gene, the elicitor molecule will not be recognized by the host, resulting in compatible interaction (susceptibility).

This theory is supported by the receptor–ligand model, which predicts that plant proteins encoded by the *R* gene directly recognize *Avr* proteins of pathogens [61] and trigger the plant defense system. It can be inferred that the ability of plants to recognize and respond to a pathogen or, in the case in question, to the methanolic extract of *R*. *guttatus* cutaneous secretions, dictates either the susceptibility or resistance of an interaction.

The effect of reduced glyceollin accumulation and increasing susceptibility demonstrated the importance of evaluating cultivars with different genetic materials. Besides their potential use for crop protection, elicitors can serve as versatile tools for selecting disease-resistant cultivars [1]. High phytoalexin-yielding cultivar may be selected for breeding as a source of resistance cultivar [58]. Expression of the non-native phytoalexin synthesis in crop plants could increase plant immunity and resistance to pests and diseases because host pathogens may have low capability to detoxify non-native phytoalexins, which reduce the rate of colonization until other parts of innate plant defense are activated to maximum levels, such as the synthesis of antimicrobial reactive oxygen species (ROS) [62].

### Synthesis of phaseolins in bean hypocotyls and deoxyanthocyanidins in sorghum mesocotyls

The methanolic extract of *R*. *marina* demonstrated the biological activity of phaseolin synthesis. However, in our study, the methanolic extract of *R*. *guttatus* did not show elicitor activities. Ferreira et al. [42] showed the differences in composition between *R*. *marina* and *R*. *guttatus* cutaneous secretions, in terms of the number and type of constituents. Telocinobufagin is found only in *R*. *marina* cutaneous secretions, together with another three bufadienolides: marinobufagin, bufalin, and resibufogenin. However, only bufadienolide (marinobufagin) was identified in *R*. *guttatus* cutaneous secretions [42, 63]. Bufadienolides are an important group of steroid hormones that has shown vasoconstriction [64, 65], antiviral [66–68], antitumor [43, 69–71], cytotoxic [42, 72], antibacterial, and antifungal agents [41, 73]. These elicitor activities could be attributed to the presence of these bufadienolides.

In this study, crude extracts were used, suggesting some type of synergism between active compounds [74]. A crude (untreated) extract from any source of natural products typically contains novel, structurally diverse chemical compounds [75], active, partially active, as well as many inactive compounds [33, 76]. Lahlou [75] cited that isolates often work differently from the original natural products, which have synergies and may combine, say, antimicrobial compounds with compounds that stimulate various pathways of the immune system. Moreover, during extraction, as well as during the isolation processes, transformation and degradation of compounds can occur [77].

In fact, studies with the use of natural products have provided a prospective strategy for identifying novel chemical structures. Natural products, containing inherently large-scale structural diversity more than synthetic compounds, have been the major resources of bioactive agents and will continually play as protagonists for discovering new chemical templates [78, 79]. Animals, plants, marine life, fungi, bacteria, and other organisms [80] are important sources of biologically active substances with structural diversity and novel mechanisms of action, which can possibly provide patentable products [35, 36, 42].

With the exception of studies on plant-based preparations, the existing literature has no information available on the mechanisms of defense in the bean plants induced by the cutaneous secretions of amphibians. Examples include reports of induction of phaseolin accumulation in beans using essential oils of *Cymbopogon citratus* and *Rosmarinus officinalis* [81] and homeopathic preparations of *C*. *citriodora* and *C*. *carbonica* [30]. Therefore, our results may facilitate the development of other studies on this subject.

In the current study, the methanolic extracts of *R*. *guttatus* and *R*. *marina* showed no deoxyanthocyanidin synthesis in sorghum mesocotyls. This suggests that the extracts analyzed do not induce the synthesis of phytoalexins in sorghum because of the lack of activation of the genes required for deoxyanthocyanidin synthesis. Recent evidence revealed the existence of many receptors with which elicitor molecules interact to induce phytoalexin synthesis [82, 83]. These receptors need to be specific to discriminate between structurally similar active and inactive molecules [84].

The mechanisms of action involved in phytoalexin synthesis by bioprospecting of secondary metabolites of animals are still unknown. However, phytoalexin synthesis in sorghum using natural compounds, including plant and basidiocarp extracts, has been reported. Meinerz et al. [85] and Peiter-Beninca et al. [86] found a strong response using basidiocarp extracts of *P*. *sanguineus* and a decoction of *Adiantum capillus-veneris*, respectively, in the induction of phytoalexin synthesis in etiolated sorghum mesocotyls. Baldin et al. [87] found that phytoalexin synthesis was induced in sorghum mesocotyls by ethanol extracts of propolis.

### Induction of enzyme activity in soybean cotyledons

In the present study, higher synthesis rate of POX was observed with high concentration of the methanolic extract of *R*. *guttatus* cutaneous secretions in the cotyledons of ‘TMG 132 RR’ (0.5 mg/mL). Reduced activities of POX were observed with lower concentrations of methanolic extract of *R*. *guttatus* cutaneous, which induced of susceptibility in the cotyledons of ‘TMG 132 RR’. In the literature, peroxidases are involved in a broad range of physiological processes throughout the plant life cycle, probably due to the high number of enzymatic isoforms (isoenzymes) and to the versatility of their enzyme-catalyzed reactions [88, 89].

Plant POX is involved in auxin metabolism, lignin and suberin formation, cross-linking of cell wall components [90], phytoalexin synthesis, and the metabolism of ROS and reactive nitrogen species [91, 92]. This suggests that the suppression of phytoalexin synthesis in ‘TMG 132 RR’, previously described in this study, can be related to reduction in the activity of these enzymes.

Thus, POX plays a crucial role in the protection of plant cells, either triggering defense reactions or overcoming the deleterious effects of oxidative stress [93]. According to Stangarlin et al. [94], changes in POX activity were correlated with resistance or susceptibility in different plant pathosystems.

The increased PPO activity in the cotyledons of ‘TMG 132 RR’ by the methanolic extract of *R*. *guttatus* cutaneous secretions reached a maximum in the higher concentrations. These results could be related to increased POX activities, which are also observed in high concentration. PPOs catalyze the oxidation of several phenols to *o*-quinones [95] and are involved in the regulation of multiple redox reactions and ROS signals in plants [96]. Thus, simultaneous increase in PPO and POX activities may act collectively in antioxidant activities, as PPO may promote POX activity in the oxidation of phenolic compounds [97]. Barghava and Sawant [98] reported that simultaneous expression of multiple antioxidant enzymes has been shown to be more effective than single expression for developing transgenic plants with enhanced tolerance to multiple environmental stresses.

In the present study, the lowest concentration of the methanolic extract of *R*. *marina* cutaneous secretions (0.1 mg/mL) produced the highest PPO activity in ‘TMG 132 RR’. A possible explanation for this result is the occurrence of the hormesis effect, a dose–response phenomenon that is characterized by stimulation of a biological response at lower doses and inhibition of this response at higher doses [99–101].

In hormesis, the dose–response relationship is biphasic and is represented as a dose–response curve in the form of an inverted “U” or “J.” In addition, the biological activity is abolished in a plasma concentration range when the dose is lower than the lower limit of the dose–response curve, but the activity is restored at much lower concentrations [99, 100]. Therefore, it is assumed that the exposure of the cotyledons of this soybean cultivar to the lowest concentration of the methanolic extract of *R*. *marina* cutaneous secretions induced PPO synthesis, whereas high doses did not produce the same effect.

Different *R*. *guttatus* methanolic extract concentrations reduce the specific activity of GLU in ‘TMG 132 RR’ and consequently inhibit defense responses. As observed, the increase in the concentrations of the extract agreed with the decrease in GLU activity. This tendency of GLU is also observed in ‘Monsoy 8372 IPRO’ treated with the methanolic extract of *R*. *marina*. Reduced GLU activity may translate into higher cotyledon susceptibility of this cultivar. Sun et al. [102] indicated that GLU accumulation is often recognized as a signal of induced resistance and has an inhibitory effect on pathogen infection.

GLU hydrolyzes β-1,3-glucan, which is a major cell wall component of many fungi, resulting in the destruction of pathogen structures or propagules [103, 104]. Furthermore, GLUs are known to release oligosaccharides from the walls of fungi, which in turn, act as signals in the elicitation of host defense responses, such as phytoalexin synthesis [30, 105]. The results, therefore, suggest the role of reduced GLU activity in the reduction observed in the synthesis of glyceollin in ‘TMG 132 RR’. Lima Melo et al. [106] reported that abiotic resistance inducers, calcium phosphite, copper phosphite, Agro-Mos, calcium silicate, Biopirol, and Bion suppressed the activity of GLU in pineapple fruits, compared with the control (sterile water), as demonstrated in this study.

The higher total protein content at lower concentrations of the methanolic extract of *R*. *guttatus* cutaneous secretions in the cotyledons of ‘Monsoy 8372 IPRO’ and ‘TMG 132 RR’ demonstrates that the substances present in the extract may cause a biphasic dose–response. This hermetic effect can be seen in the total protein content and activity of GLU in ‘TMG 132 RR’. This response, as mentioned before, showed that a biological response is stimulated at low doses of a compound and inhibited at high doses of the same compound [99]. The reduction in the biochemical mechanism in treated cotyledons of this cultivar may be related to the resumption of homeostatic balance, which would require less synthesis of defensive compounds and consequently lower metabolic cost due the balance in plants [107]. This metabolic cost can result in disfavoring the primary pathways for the synthesis of defense compounds, resulting from the activation of latent defense mechanisms [108].

In the present study, the effect of the evaluated extracts on the induction of defense responses was distinct in ‘TMG 132 RR’ and ‘Monsoy 8372 IPRO’. ‘TMG 132 RR’ demonstrated higher responses. In contrast, nontransgenic ‘TMG 4182’ did not respond to the extracts. It has, therefore, become evident that this result may be related to the genotype of these cultivars. Walters et al. [23] reported that the host genotype is known to affect the expression of induced resistance. The authors suggested that defense responses can be influenced by the domestication of some plants [23, 109].

Although considerable progress has been made to understand plant defense responses, very little is known about the role of primary metabolic pathways required for growth and development in regulating plant defense responses [110]. According to the authors, in spite of evidence in plant defense responses, more studies are needed to identify additional components involved in defense responses as well as detailed characterization of the mechanisms underlying such responses.

Besides, the present study is the first to report elicitation of defense responses produced by cutaneous secretions of amphibian species. Thus, further research is also necessary to understand the bioactivity presented by the extracts and to discover if their bioactivities are related to the result of synergistic interactions among several components present in the crude extract or if a fractioned component is responsible for this action.

## Conclusions

Amphibian species the family Bufonidae have recently emerged as powerful tools to provide a rich source of secondary metabolites that can be useful as new chemical templates for drug discovery. In the last decades, many discoveries of novel chemical structures and biological mechanisms that can be applied for the development of disease and pest control agents in agrochemical research have had their origin in a wide range of natural products from a variety of sources. This study established that crude extracts of cutaneous secretions of *R*. *guttatus* and *R*. *marina* are capable of promoting bioactivity in some defense responses in soybean cultivars and bean. Thus, the methanolic extract of the cutaneous secretions of *R*. *guttatus* suppressed glyceollin synthesis and GLU activity in ‘TMG 132 RR’. In ‘TMG 132 RR’, the extract induced POX and PPO activities at higher concentrations. In the same cultivar, higher total protein content was observed at the concentration of 0.2 mg/mL. In ‘Monsoy 8372 IPRO’, the lower concentration of this extract (0.1 mg/mL) increased total protein content. For the extract of the *R*. *marina* species, glyceollin synthesis was induced at lower concentrations in ‘Monsoy 8372 IPRO’ and ‘TMG 132 RR’. Minor concentrations of this extract also induced phaseolin synthesis in beans. Regarding enzymatic activity, induction of POX activity was observed at higher concentrations in ‘Monsoy 8372 IPRO’ (0.5 mg/mL). In this same cultivar, PPO and GLU activities were reduced in the presence of this extract. In ‘TMG 132 RR’, higher PPO activity was observed at a lower concentration (0.1 mg/mL). The study in soybean suggests that the bioactivity in defense responses is influenced by cultivar genotypes. In sorghum mesocotyls, the extracts did not express bioactivity.

In summary, we consider that this study provided relevant information for new opportunities of assays with components derived from secondary metabolites of amphibian species from southern Amazon, the effects of which may supplement the continuing need of new control agents of disease and pest. Just as important as this, we consider that studies with species native to southern Amazon may, in a way, contribute to the preservation of these resources. Unfortunately, as a result of ongoing climate changes and anthropogenic factors, a significant decrease in global amphibian species in the next years is predicted, endangering the sources of potential new molecules from nature.

## Supporting information

S1 Table Synthesis of glyceollins in soybean cotyledons treated with different concentrations of *R. guttatus* and *R. marina* (0.1, 0.2, 0.3, 0.4, and 0.5 mg/mL), sterile water (0), and *Saccharomyces cerevisiae* (SC). (XLSX)

S2 Table Synthesis of phaseolins in bean hypocotyls and deoxyanthocyanidins in sorghum mesocotyls treated with different concentrations of *R. guttatus* and *R. marina* (0.1, 0.2, 0.3, 0.4, and 0.5 mg/mL), sterile water (0), and *Saccharomyces cerevisiae* (SC). (XLSX)

S3 Table Enzyme activity in ‘Monsoy 8372 IPRO,’ ‘TMG 132 RR,’ and nontransgenic cultivar ‘TMG 4182’ treated with different concentrations of *R. guttatus* (0.1, 0.2, 0.3, 0.4, and 0.5 mg/mL), sterile water (0), and *Saccharomyces cerevisiae* (SC). (XLSX)

S4 Table Enzyme activity in ‘Monsoy 8372 IPRO,’ ‘TMG 132 RR,’ and nontransgenic cultivar ‘TMG 4182’ treated with different concentrations of *R. marina* (0.1, 0.2, 0.3, 0.4, and 0.5 mg/mL), sterile water (0), and *Saccharomyces cerevisiae* (SC). (XLSX)
